# Agentic systems in computational pathology: architectures, evidence, and translational challenges

**DOI:** 10.1186/s12967-026-08829-0

**Published:** 2026-08-28

**Authors:** Xinyu Lu, Qiankun Li, Yakun Gao, Wei Dong, Mengyao Lyu, Siyuan Ma, Jinyue Li, Yufeng Wu, Linghan Cai, Tianyi Zhang, Shangqing Lyu, Zeyu Liu, Hui Liu, Susu Luo

**Affiliations:** 1 Third Department of Hepatic Surgery, Shanghai Eastern Hepatobiliary Surgery Hospital, Shanghai, 200438 China; 2 College of Computing and Data Science, Nanyang Technological University, Singapore, 639798 Singapore; 3 Department of Plastic Surgery, Huashan Hospital, Fudan University, Shanghai, 200040 China; 4 PuzzleLogic Pte Ltd, 1 Claymore Drive, Singapore, 229594 Singapore; 5 Department of Electrical and Computer Engineering, National University of Singapore, Singapore, 117583 Singapore; 6 Moores Cancer Center, University of California San Diego, La Jolla, CA 92037 USA

**Keywords:** Agentic artificial intelligence, Digital pathology, Whole-slide imaging, Large language models

## Abstract

**Background:**

Digital pathology supports whole-slide imaging, remote review, and computational analysis. Most pathology AI systems, however, remain restricted to predefined tasks. Agentic architectures coordinate perception models, language-based reasoning, external tools, and feedback-dependent actions, but their clinical evidence is derived mainly from retrospective benchmarks and research prototypes.

**Main body:**

We review agentic systems in computational pathology using an operational taxonomy based on dynamic control flow, inference-time tool selection, and knowledge integration. We assess architectures, enabling technologies, and applications in diagnosis, prognosis, and therapeutic support. Reported gains are difficult to attribute to agentic organization because studies differ in backbones, training data, and inference budgets. We therefore emphasize validation scope, computational cost, workflow integration, hallucination and security risks, regulatory requirements, patient preferences, and the conditions under which specialist non-agentic models remain preferable.

**Conclusions:**

Agentic architectures have established technical feasibility, but not clinical benefit. Translation should prioritize verifiable tasks, matched comparisons, prospective and external validation, lifecycle governance, and interfaces that preserve pathologist oversight.

**Graphical Abstract:**

**Figure:**
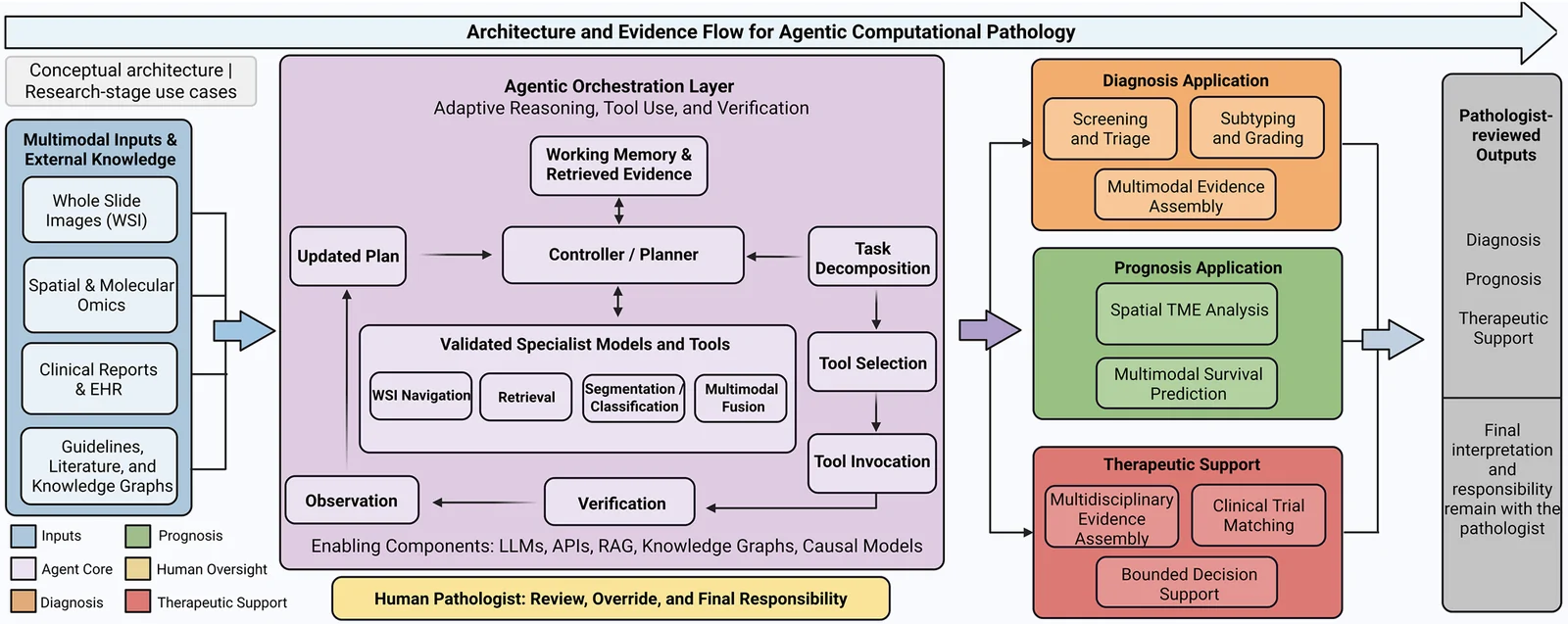

## Introduction: pathology at the crossroads

Digital pathology is implemented at different scales across institutions, from external consultation and satellite sign-out to end-to-end digitization of pathology workflows [1]. Here, digital pathology denotes the infrastructure and workflows used to digitize, store, review, and manage slides and associated metadata, whereas computational pathology denotes the quantitative extraction and integration of information from digitized pathology images and associated clinical or molecular data [2]. Whole-slide imaging converts tissue sections into high-resolution digital images [3] and supports the transition from microscopy to digital workflows [1]. In a large regional laboratory in the Netherlands, digital implementation saved more than 19 hours of logistics time per working day, equivalent to 2.63 full-time employees [4]. As a single-site workflow analysis, this study demonstrates potential logistical efficiency but does not by itself establish a general solution to workforce shortages.

Digital adoption also creates practical challenges. Digital review can take longer than glass-slide review for some tasks [5]. At high magnification, pathologists may lose spatial context and spend additional time navigating gigapixel WSIs [6]. Diagnostic review therefore proceeds across scales, from low-magnification screening to targeted inspection and synthesis across regions [7, 8]. These demands motivate navigation and decision-support tools, but they do not establish that manual diagnosis is globally unsustainable.

Digital workflows enabled the use of artificial intelligence (AI) for pathology image analysis [2, 9]. Early work, approximately 2015–2020, centered on convolutional neural networks and multiple-instance learning [10–14]. From 2021 onward, foundation models and multimodal learning became prominent [15–19]. Quilt-1 M provided large-scale image-text pairs at patch level [20]; PathAlign supported slide-level report generation [21]; and PathChat combined histology images with language-based interaction [22]. Virchow, trained on 1.5 million WSIs, reported a pan-cancer detection AUC of 0.95 [23]. A foundation model is broadly pretrained and adaptable to multiple downstream tasks, but it is not inherently agentic. Agentic systems can be further classified by operational scope: generalist agents operate across multiple task domains, whereas specialist agents are limited to predefined pathology tasks and bounded tool sets.

Despite strong benchmark performance, current models have limitations. Their internal representations rarely provide a faithful account of how a prediction was produced [24]. Fixed inference graphs do not reproduce the case-dependent sequence of observation, hypothesis generation, and verification used in diagnostic practice [24, 25]. Many MLLMs also operate on selected patches rather than navigating a complete gigapixel WSI [25]. Multimodal gains vary by cancer type [26, 27], and independent external validation remains limited [28]. These limitations concern transparency, task adaptability, and transportability, but do not imply that fixed models are inherently unsuitable for clinical use.

Recent agent research offers one way to coordinate adaptive analysis [29, 30]. Related systems have been studied in web navigation [31], task automation [32, 33], multimodal reasoning [34], and robotic interaction [35]. Here, an agentic system is one in which intermediate observations can alter subsequent control flow, tool use, or evidence retrieval [36]. Generative-agent architectures, for example, combine observation, memory retrieval, reflection, planning, and action [29]. The degree of adaptivity should be assessed from inference behavior rather than system labels.

Representative work in agentic systems includes WebGPT and WebShop for web-based information retrieval [31, 37], ReAct for interleaving reasoning with actions [38], and CPathAgent for region-guided analysis of high-resolution pathology images [36]. In medicine, multi-agent systems have been evaluated for collaborative reasoning and diagnostic tasks on retrospective benchmarks [39–41]. Existing systems range from task-specific tool-using agents to broadly scoped medical assistants. Broad medical coverage, however, does not establish pathology-specific capability, which remains insufficiently evaluated [42]. Pathology-focused research has begun to address WSI navigation, diagnostic reasoning, and workflow automation, but the evidence is dispersed across heterogeneous tasks and architectures. This review therefore maps agentic systems in computational pathology, examines their architectures and clinical evidence, and identifies requirements for clinically evaluated deployment.

In this review, we synthesize agentic systems for diagnosis, prognosis, and therapeutic decision support. We make three contributions. First, we apply an operational criterion that distinguishes adaptive reasoning systems from modular pipelines. Second, we relate reported capabilities to validation, cost, workflow, security, regulatory, and workforce requirements. Third, we identify where specialist non-agentic models remain preferable. Because the literature consists mainly of retrospective research prototypes, these contributions concern architecture and evidence requirements rather than established clinical benefit. We identified no system in this review with regulatory clearance for autonomous diagnostic use. Agentic systems are therefore discussed as decision-support tools, with pathologists retaining final interpretive responsibility.

## Agent systems: architecture and core capabilities

### From AI tools to agent systems in digital pathology workflow

Pathologists typically examine a slide across scales, first assessing tissue architecture at low magnification, then inspecting selected regions at higher magnification, and finally integrating evidence across regions [43]. This process is iterative and case dependent. Exact magnification choices vary by specimen, task, and individual practice.

Specialist CNN and multiple-instance learning (MIL) models can perform bounded tasks such as tumour detection or tissue classification and may achieve performance comparable to that of pathologists on selected benchmarks [44]. Conventional MIL systems generally aggregate predefined tiles or patches without slide-level adaptive navigation [45]. By contrast, PathAgent uses a fixed Navigator-Perceptor-Executor module graph, but its region selection and iterative self-evaluation steps can vary across cases [46]. Recent multimodal agents can similarly select successive image crops in response to intermediate observations [47]. Such adaptive control may make intermediate actions more inspectable, but it does not guarantee faithful interpretability, because post hoc explanations may not accurately represent the computation that produced a prediction [48, 49].

**Fig. 1:**
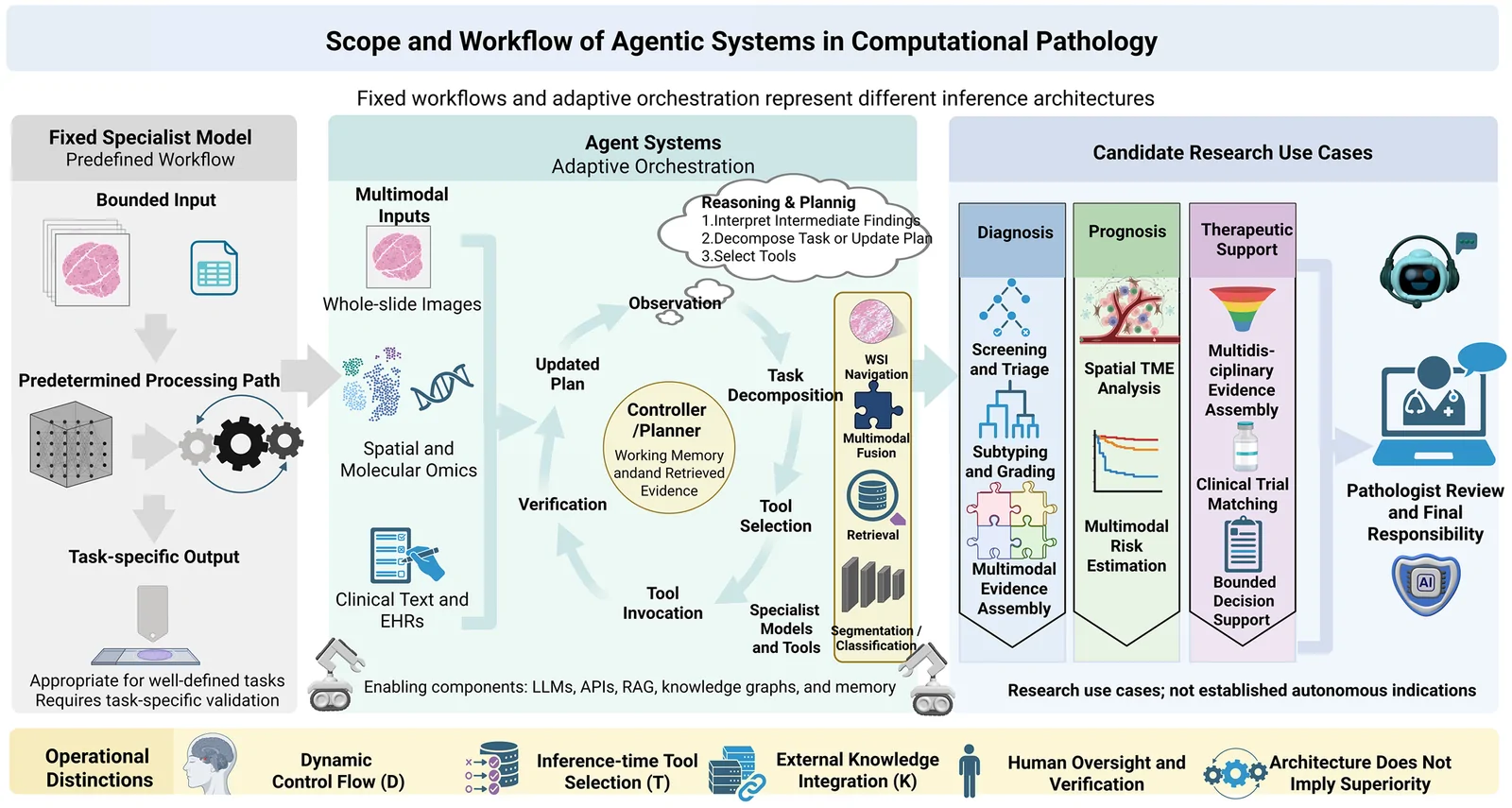
Scope and workflow of agentic systems in computational pathology. Defines the scope of this review. Fixed specialist systems map bounded inputs to task-specific outputs through a predetermined workflow, whereas agentic systems may use intermediate findings to update plans, select tools, invoke specialist models, or retrieve external knowledge. The lower strip identifies dynamic control flow (**D**), inference-time tool selection (**T**), and external knowledge integration (**K**). The right panel groups candidate research use cases into diagnosis, prognosis, and therapeutic support. These are not established indications for autonomous clinical practice, and architecture alone does not imply superiority. Created with BioRender.com

Some agentic systems coordinate sequential analyses through iterative perception, reasoning, and action [36, 46, 50]. They may vary where to examine, when to request another view, or which tool to invoke. Such adaptivity is an architectural property. Its clinical value requires matched comparison with validated specialist models (Fig. 1).

### Multi-agent architectures and developmental stages

Gao et al. [51] describe four core components of biomedical AI agents: perception, interaction, memory, and reasoning, and propose a Level 0–3 autonomy scale. Level 0 is non-agentic, Level 1 executes a human-specified task, Level 2 determines case-specific subtasks and selects tools, and Level 3 generates hypotheses or methods. Most systems reviewed here operate at Level 1 or the Level 1–2 boundary. We assign Level 2 only when task decomposition and tool selection vary at inference. The operational criteria used for these assignments are defined in Sect. “Fundamental distinctions between agent systems and traditional methodologies”.

Medical-image multi-agent systems remain technically heterogeneous. In a systematic review, 84.8% used empirical parameter calibration, 36.4% required domain knowledge, 27.3% were not fully automated, and 72.7% relied on simple reactive agents [52]. These findings describe the surveyed literature rather than a progression toward higher autonomy. They also indicate why task decomposition, tool use, and knowledge integration must be reported explicitly.

WSI-Agents uses fixed task-allocation, verification, and summarization stages [53]. On WSI-Bench and WSI-VQA [54], it reported scores 10–17% higher than WSI-LLaVA [55], MDAgents [41], and Med-Agents [39]. Because these baselines differ in backbones, training data, and inference budgets, the comparison does not isolate multi-agent organization [53]. Luo et al. [56] describe complementary design choices for memory, planning, action, and centralized or decentralized collaboration. Renal-Agent [57] coordinates vision-language, segmentation, and classification models through a central language model. Its conference abstract reported a mean quadratic-weighted kappa of 0.73 across 16 Banff scores and balanced accuracy of 0.97 for rejection-subtype classification. The reported cohorts contained 15,000 MGB biopsies and 1,200 AUSCAD biopsies. These retrospective results do not establish clinical utility.

These studies show that task decomposition, specialist tools, and verification can be combined in pathology research systems. They do not yet establish clinical-grade autonomy: most evaluations are retrospective, component-matched ablations are uncommon, and workflow benefit has not been tested prospectively.

### Fundamental distinctions between agent systems and traditional methodologies

Because “agent” is used inconsistently, we classify systems along three dimensions. Dynamic control flow (D) requires inference steps to change in response to intermediate findings. Inference-time tool selection (T) requires the reasoning module, rather than a fixed pipeline, to choose an external model or resource. Knowledge integration (K) requires retrieval and use of information beyond the primary input. Table 1 summarizes the operational classes. Each dimension is coded as Yes, Partial, or No and does not itself imply clinical superiority. Tables 2 and 3 then apply this framework, report autonomy under Gao et al. [51] and identify contestable assignments.

**Table 1:** Operational taxonomy of computational pathology systems

System class	D	T	K	Typical autonomy	Representative systems
Perception or fixed model	No	No	No	0	Virchow; UNI; PAMA; PathChat; CLAM-type MIL
Reasoning agent	Partial/Yes	Partial/Yes	Optional	1–2	PathAgent; Ferber Clinical Agent; AgentMRI; Pathology-o3
Workflow orchestration system	Partial/Yes	Partial/Yes	Usually Yes	1–2	TissueLab; TxAgent; Renal-Agent; WSI-Agents
Boundary rule	Intermediate findings alter subsequent steps	Reasoner selects a tool at inference	Retrieves evidence beyond the primary input	Architecture only	Labels and benchmark scores do not determine class

Abbreviations: D, dynamic control flow; T, inference-time tool selection; K, external knowledge integration; CLAM, clustering-constrained attention multiple instance learning; MIL, multiple instance learning; WSI, whole-slide image

Autonomy levels: Level 0 represents non-agentic stage (utilizing only ML tools); Level 1 agents execute narrowly-defined tasks specified by humans; Level 2 agents autonomously define task sequences and invoke expansive tool sets (current developmental objective for pathology agents); Level 3 agents possess the capacity to generate innovative hypotheses and develop novel methodologies

**Table 2:** Operational classification and validation status of systems described as agentic or agent-assisted

System	Citation	Operational class	D	T	K	Autonomy	Publication status	Validation scope	Reported inference cost
PathAgent	[46]	Reasoning agent	Partial	Partial	No	1–2	Preprint	Retrospective VQA benchmarks; no prospective clinical cohort	Not reported
TissueLab	[58]	Workflow orchestration system	Yes	Yes	Yes	2	Preprint	Multiple retrospective or open datasets; clinician-feedback experiments	Workflow generation < 60 min and feedback update < 10 s; distributed multi-GPU servers mentioned; standardized per-case cost not reported
Biomni	[59]	Generalist workflow agent	Yes	Yes	Yes	2	Journal article	Heterogeneous biomedical benchmarks and research demonstrations; no prospective pathology cohort	Not reported
PathChat	[22]	Multimodal assistant and comparator	No	No	No	0	Journal article	Curated PathQABench; no WSI navigation or clinical workflow trial	Not reported
CPathAgent	[36]	Navigation/reasoning model	Partial	No	No	1	Conference paper	PathMMU, PathMMU-HR2, and six TCGA datasets; retrospective	Not reported
SurvAgent	[60]	Fixed multimodal inference workflow	Partial	No	Yes	1	Preprint	Five TCGA cohorts; internal retrospective evaluation	No extra training reported; inference used 4 ×RTX A6000 (48 GB). Latency and monetary cost not reported
Ferber Clinical Agent	[61]	Tool-augmented reasoning agent	Partial	Yes	Yes	1–2	Journal article	Twenty simulated multimodal cases with blinded expert scoring	Not reported
MEREDITH	[62]	Fixed multi-agent RAG workflow	No	No	Yes	1	Journal article	Ten fictitious cases and 59 variant assessments	Not reported
Multimodal LLM-Agent Framework	[63]	RAG-enabled LLM decision-support workflow	No	No	Yes	1	Journal article	Retrospective development cohort and 105-case blinded evaluation	Pathology-model training used RTX 4090 (24 GB), i9 CPU, and 128 GB RAM; agent inference latency and cost not reported
TxAgent	[64]	Tool-augmented reasoning agent	Yes	Yes	Yes	2	Preprint	DrugPC and TreatmentPC benchmark tasks	Not reported
Healthcare Agent Orchestrator	[65]	Workflow orchestration system	Partial	Yes	Yes	1–2	Preprint	Seventy-one de-identified oncology cases; technical evaluation	Not reported
Multi-Agent AI Platform	[66]	Neuro-symbolic multi-agent platform	NR	NR	Yes	NR	Journal article	Prospective operational evaluation in 3,804 patients; held-out patient-trial pairs with dual-oncologist adjudication	Median approximately 30 min per patient, including automated processing and clinical review; hardware and monetary cost not reported
WSI-Agents	[53]	Fixed multi-agent workflow	No	Yes	Yes	1	Conference paper	WSI-Bench and WSI-VQA; retrospective benchmark comparison	Not reported
Renal-Agent	[57]	Workflow orchestration system	Yes	Yes	Yes	2	Conference abstract	Retrospective MGB cohort and external AUSCAD cohort	Not reported
AgentMRI	[67]	Reasoning agent	Partial	Yes	No	1–2	Journal article	Synthetic corruption tasks with an independent test set	28.48–36.99 s per case; i7/64 GB/Quadro P2200 plus cloud APIs; monetary cost not reported
AutoBA	[68]	Workflow agent	Partial	Yes	Partial	1–2	Journal article	Forty multi-omics analysis scenarios	3–18 min excluding code execution; A100 workstation, 252 GB RAM, 112 CPU cores; model memory 12.55–74 GB; monetary cost not reported
Pathology-o3	[69]	Navigation agent	Partial	Partial	No	1–2	Journal article	Internal Stanford cohort and one external lymph-node cohort	US$0.02–0.12 per slide and 5.8–13.4 s per image for Gemini variants; hardware not reported
STAgent	[70]	Spatial-omics workflow agent	Partial	Yes	Yes	1–2	Preprint	Single spatial-transcriptomics biological study	Described as minutes; standardized latency and cost not reported
PhenoGraph	[71]	Multi-agent workflow	Yes	Yes	Yes	2	Preprint	Two retrospective spatial-transcriptomics datasets	Not reported

Notes: Partial indicates that the criterion is satisfied only during some inference stages or remains constrained by a predefined workflow. D, T, K, and autonomy describe the reported inference architecture rather than clinical efficacy. Reported inference costs are reproduced as provided by the original studies and are not directly comparable when hardware, latency definitions, or accounting methods differ

Abbreviations: AI, artificial intelligence; D, dynamic control flow; T, inference-time tool selection; K, external knowledge integration; NR, not reported; LLM, large language model; WSI, whole-slide image; VQA, visual question answering; RAG, retrieval-augmented generation; TCGA, The Cancer Genome Atlas; MGB, Mass General Brigham; AUSCAD, Australian Chronic Allograft Dysfunction; API, application programming interface; GPU, graphics processing unit; CPU, central processing unit; RAM, random-access memory; GB, gigabyte

**Table 3:** Non-agentic comparators and reasons for classification

System	Citation	Task	Operational class	D	T	K	Validation scope	Why non-agentic
Graham colon-biopsy screening algorithm	[72]	Normal versus abnormal screening	Specialist classifier	No	No	No	Four-centre retrospective validation	Threshold is configured at deployment; no inference-time tool or knowledge selection
Glioma classification system	[73]	WHO 2021 glioma classification	Foundation-model MIL pipeline	No	No	No	Two external cohorts	Inference graph and label space are fixed
PDAC hybrid framework	[74]	Subclonal simulation and risk stratification	Agent-based biological simulation plus classifier	No	No	No	Simulation study with retrospective TCGA calibration	Cell-level simulation is dynamic but does not select reasoning steps, tools, or knowledge at inference
Prostate cancer GCN grading	[75]	Gleason grade-group classification	Specialist graph model	No	No	No	Public datasets and small private validation set	Predetermined graph construction and classifier
stKeep	[76]	Spatial tumour-microenvironment analysis	Fixed heterogeneous graph model	No	No	No	Multiple retrospective spatial datasets	Graph topology and aggregation path are fixed before inference
Lunit SCOPE IO	[77]	Immune-phenotype quantification	Specialist segmentation and classifier	No	No	No	Single retrospective resection cohort	Bounded fixed workflow without inference-time branching
LLM-guided multimodal MIL	[78]	Five-year lung-cancer survival	Fixed multimodal model	No	No	No	Six hospitals with an external validation cohort	Human-guided prompts and a predetermined fusion pipeline
MUSK	[79]	Retrieval, VQA, recurrence, and prognosis	Multimodal foundation model	No	No	No	Multiple retrospective cohorts	Fixed pretrained representation and downstream heads
CATfusion	[80]	Pan-cancer survival prediction	Fixed multimodal fusion model	No	No	No	TCGA with one CPTAC cohort	Fusion path and modality weights are learned but not selected by reasoning at inference
Brim	[81]	Pan-cancer survival prediction	Fixed multimodal fusion model	No	No	No	TCGA and one colorectal cohort	Predetermined bridging architecture without tool use
Claude spatial analysis	[82]	Spatial-transcriptomics interpretation	LLM-assisted workflow	No	No	No	Single proof-of-concept tissue sample	Human directs the workflow; no autonomous planning or tool selection
General LLM evaluation	[83]	Breast-cancer treatment recommendations	Language-model baseline	No	No	No	Single-centre retrospective case set	Prompt-to-output inference without external tools or retrieval
PRISM and OncoLLM	[84]	Clinical-trial matching	Fixed RAG pipeline	No	No	Yes	Retrospective patient-trial pairs	Retrieval and ranking sequence are predefined
Morpheus	[85]	Counterfactual perturbation design	Optimization model	No	No	No	Retrospective cohorts with in vitro validation	Fixed optimization objective without agentic control flow

Note: Classification is based on the operational D/T/K criteria. The designation “non-agentic” describes inference architecture and does not imply inferior predictive performance, clinical value, or methodological quality

Abbreviations: D, dynamic control flow; T, inference-time tool selection; K, external knowledge integration; MIL, multiple instance learning; WHO, World Health Organization; PDAC, pancreatic ductal adenocarcinoma; TCGA, The Cancer Genome Atlas; GCN, graph convolutional network; VQA, visual question answering; LLM, large language model; RAG, retrieval-augmented generation; CPTAC, Clinical Proteomic Tumor Analysis Consortium

Agentic and fixed systems differ in how inference is controlled, not in a presumed hierarchy of capability. Foundation models such as Virchow and PAMA use predetermined encoding and downstream prediction pathways [23, 90]. Under this review’s criteria, they have no dynamic control flow, inference-time tool selection, or external knowledge retrieval. An agentic system qualifies only when intermediate findings alter at least one of these operations.

An “agent” label does not establish agentic reasoning. Tan et al. used multi-agent reinforcement learning for metabolomic feature selection and reported 94.9% accuracy in a small-sample setting [91]. The feature selectors do not plan, invoke tools, or retrieve knowledge during inference. PolyPath [92] and a multimodal HNSCC prognostic model [93] likewise process multiple inputs through fixed graphs. Such models may serve as specialist components within an agentic workflow, but they remain non-agentic under the D/T/K criteria.

### Key enabling technologies

**Fig. 2:**
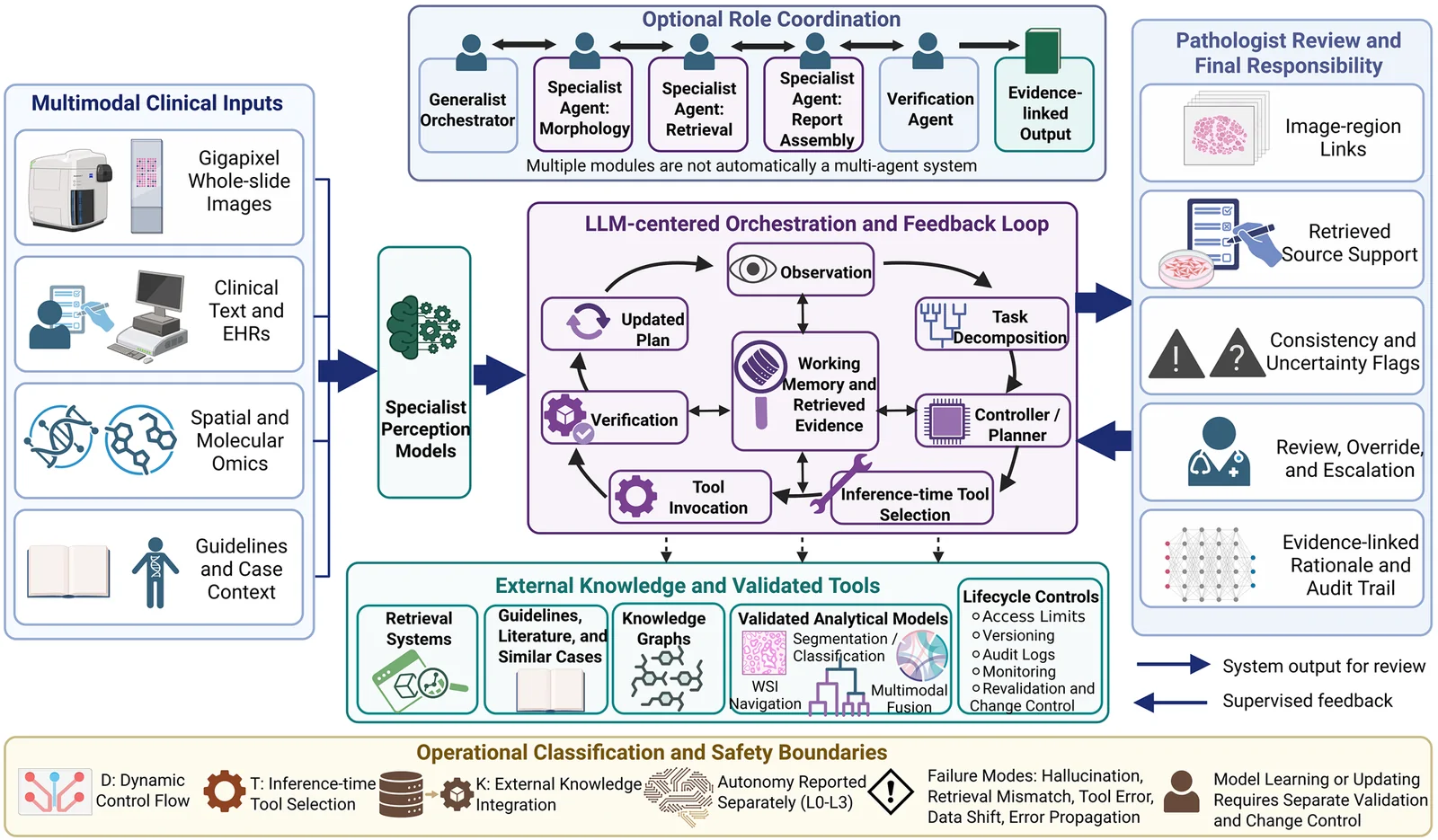
Architecture and enabling components of agentic systems in computational pathology. Depicts an LLM-centered orchestration and feedback architecture. Multimodal inputs are processed by specialist perception models, while the controller/planner decomposes tasks, selects and invokes tools, retrieves evidence, verifies outputs, and updates the plan. Optional role coordination may involve generalist, specialist, and verification agents; multiple modules alone do not constitute a multi-agent system. External resources include retrieval systems, guidelines, knowledge graphs, and validated analytical models. Outputs are linked to image regions and retrieved sources for pathologist review. Dynamic control flow (**D**), inference-time tool selection (**T**), external knowledge integration (**K**), and reported autonomy are distinct properties; model learning or updating requires separate validation and change control. Created with BioRender.com

Agentic systems also depend on enabling technologies that support four functions. LLMs and natural language processing support decision-making; APIs enable external tool use; RAG and knowledge graphs provide external knowledge; and causal methods support inference and data augmentation (Fig. 2).

**Tool utilization and external model invocation.** Toolformer showed that language models can learn to call external APIs [33]. In pathology, an orchestrator can invoke specialist modules for segmentation, cell counting, or biomarker prediction. In 20 realistic simulated oncology cases, Ferber et al. [61] reported that adding specialist tools increased the proportion of expected answers supplied from 30.3% to 87.2%. This proof-of-concept did not isolate orchestration from tool quality or establish clinical effectiveness.

**Large language models and natural language processing.** LLMs can process unstructured pathology text for report parsing, terminology normalization, and structured extraction. Prompting may reduce annotation requirements, but performance remains task- and model-dependent. Huang et al. [94] reported 89% accuracy for TNM extraction from more than 900 reports at a reported cost below US$10.

**Retrieval-augmented generation (RAG)** allows a model to retrieve external evidence during inference [95]. Jabal et al. [96] reported improved performance for complex pathology-report processing, whereas benefits were smaller for concise reports. Hu et al. [97] used retrieval of similar cases to improve report generation. Retrieval does not eliminate fabrication: output quality depends on the relevance, currency, and consistency of retrieved material. In a human audit of four generative search systems, only about half of generated sentences were fully supported, and approximately one-quarter of citations did not entail the associated claim [98]. Pathology agents should therefore verify generated claims against the retrieved passage itself, not merely the presence of a citation.

**Knowledge-graph-enhanced architecture.** Knowledge graphs encode entities and relationships in structured form [99]. They can support multi-hop queries and make retrieved paths inspectable, but graph paths do not by themselves establish causality. ESCARGOT [100] combines language-model planning, a graph-of-thought representation, and Cypher queries. On an Alzheimer’s disease knowledge-graph benchmark, it reported 85.8% accuracy on open-ended two-hop questions compared with 23.1% for the authors’ RAG baseline. This benchmark does not establish performance in pathology or clinical reasoning.

**Causal inference and data augmentation.** Causal models can separate assumed causal relations from statistical associations and generate counterfactual data under an explicit structural model [101]. Their validity depends on whether the assumed graph and interventions are credible and transport across sites. Roschewitz et al. [102] used deep structural causal models to generate cross-device counterfactual medical images and reported ROC-AUC gains of 2–6 percentage points in few-shot experiments. These are methodological results, not evidence that causal augmentation improves clinical deployment of agent systems.

**Advanced visual perception and foundation models.** Vision foundation models provide representations that an agent can use as specialist perception outputs. Virchow was trained on approximately 1.5 million WSIs and reported pan-cancer and rare-cancer detection AUCs of 0.950 and 0.937, respectively [23]. UNI, pretrained on more than 100 million tissue patches, reported 96.3% accuracy for NSCLC subtyping in an external CPTAC cohort [103]. These retrospective task results do not show that embedding the models within an agent improves performance. Deployment also remains sensitive to domain shift and gigapixel-scale compute.

These technologies have distinct limitations. LLMs can misinterpret specialized terminology and staging rules [94]; RAG provides little benefit for concise reports [96]; and knowledge-graph systems depend on graph completeness, despite strong results for ESCARGOT on a specific multi-hop benchmark [100]. Gains from causal augmentation depend on the assumed causal structure [102]. Robust systems should combine these components selectively and evaluate each under its intended operating conditions.

### Generalist versus specialist agent systems

Generalist and specialist agents describe the breadth of intended tasks, knowledge, and permitted tools. Biomni is a general-purpose biomedical AI agent that assembles tools, databases, and protocols across 25 subdomains [59]. The reported environment includes 150 tools, 105 software packages, and 59 databases, with code-based actions that support loops and conditional execution. Its breadth does not establish pathology-specific depth, and the study did not include a prospective pathology cohort. Specialist agents instead restrict tasks and tools, which can simplify validation but does not guarantee higher accuracy.

Agentic designs outside pathology illustrate complementary trade-offs. AgentMRI identifies image degradation and selects correction models; fine-tuning reduced its reported ECE from 0.248 to 0.069 [67]. AutoBA reported 90% planning success and 87.5% end-to-end execution across 40 multi-omics scenarios [68]. Both are technical evaluations. They motivate modular ecosystems but do not establish that general reasoning plus specialist tools is clinically optimal.

These examples suggest a modular design in which a general-purpose reasoning layer handles planning, tool orchestration, and uncertainty, while pathology-specific models, ontologies, and scoring tools provide domain expertise. This remains a design hypothesis rather than a demonstrated optimum and should be tested against matched specialist systems using equivalent data, backbones, and inference budgets.

### Human-AI interaction and intelligent navigation

Navigation interfaces shape how pathologists inspect WSIs [6, 104–106]. Overview-plus-detail interfaces combine panning and zooming [107–109], but they can impose cognitive and time burdens [6]. The focus-plus-context interface proposed by Jessup et al. [110] magnifies the screen centre while retaining surrounding spatial context. Agent interfaces should be evaluated for navigation errors, review time, and compatibility with routine viewers.

**Operational integration and interaction design.** A study of eight European laboratories found that laboratory information system integration required high-speed whole-slide image access, multi-user functionality, and communication with scanners and storage, while non-standard interfaces impeded integration with staining, labeling, and tracking systems [111]. An agent also requires governed read access to prior reports and ancillary results, together with a defined write path for outputs and audit logs. The most defensible interface presents an evidence-anchored summary linked to image regions and retrieved passages, with a structured action and evidence log available on demand. Review time and error correction should be measured prospectively because exhaustive review of every intermediate step may offset efficiency gains. During review, the pathologist should be able to accept, amend, reject, or escalate each proposed conclusion. The selected disposition, reviewer identity, timestamp, supporting evidence, and any amendment should be written back to the LIS or reporting system and retained in the audit record. Corrected or rejected outputs should enter model learning only through a separately governed and validated change-control process.

Human-AI collaboration can improve consistency or workload in selected tasks [6, 112, 113], and pathologists and AI may show complementary error profiles [14, 114]. In interviews with 16 pathologists, Swillens et al. [115] reported that 87% were willing to use computational pathology as decision support and 44% raised deskilling concerns. The findings motivate evaluation of assistive interfaces with explicit oversight and training, but do not establish acceptability at scale.

Pathology-CoT used an AI Session Recorder to convert continuous browsing into discrete commands and ROI boxes [69]. The training corpus comprised 10.6 hours from eight pathologists and 5,222 dialogue rounds with expert-verified labels. On colorectal lymph-node metastasis detection, Pathology-o3 reported 84.5% precision and 100% recall, compared with 46.7% and 87.5% for OpenAI o3. External recall was 97.6%. These retrospective results support behavioral supervision as a research strategy but do not establish prospective workflow benefit.

### Benchmarks relevant to pathology agent systems

Relevant benchmarks measure different units and tasks. Camelyon16 evaluates slide-level metastasis detection [86], whereas WSI-Caption assesses report-style captioning [88]. WSI-VQA tests slide-level question answering [54], PathVQA uses patch-level textbook images [87], and PathMMU aggregates expert-validated questions from diverse pathology sources [89]. These datasets support technical comparison but do not measure prospective workflow benefit, calibration under distribution shift, or patient outcomes. Table 4 summarizes these limitations.

**Table 4:** Scope and translational limitations of common pathology AI benchmarks

Benchmark	Unit and task	Ground truth	Validation scope	Clinical endpoint	Principal limitation
Camelyon16 [86]	WSI metastasis detection	Expert slide labels and lesion annotations	Multicentre retrospective challenge data	No prospective clinical endpoint assessed	Bounded detection task; does not test planning or tool use
PathVQA [87]	Patch-level visual question answering	Textbook image-question pairs	Curated test split	No prospective clinical endpoint assessed	Patch scale and question distribution differ from routine sign-out
WSI-VQA [54]	Slide-level visual question answering	Curated slide-level questions and answers	Retrospective benchmark	No prospective clinical endpoint assessed	Does not measure navigation safety or workflow benefit
WSI-Caption [88]	Slide-level report generation	Paired slide and report text	Retrospective benchmark	No prospective clinical endpoint assessed	Text similarity may not reflect factual completeness or clinical actionability
PathMMU [89]	Multisource pathology VQA	Expert-validated questions	Heterogeneous benchmark sources	No prospective clinical endpoint assessed	Case mix and question format differ from prospective practice
SlideBench-VQA [17]	WSI visual question answering	Curated slide-level questions	Retrospective benchmark	No prospective clinical endpoint assessed	Does not isolate control flow from backbone and training-data differences

Note: The listed limitations are specific to the use of these benchmarks for evaluating agentic control, workflow benefit, and clinical translation. They do not indicate that the benchmarks are unsuitable for the technical tasks for which they were originally designed

Abbreviations: WSI, whole-slide image; VQA, visual question answering

### When agentic architecture is and is not warranted

The relevant question is not whether agentic systems represent a later generation of AI, but whether a task requires adaptive orchestration. Added capability should be weighed against latency, cost, security exposure, and validation burden.

Three lines of evidence are relevant. A medical-task benchmark found that multi-agent collaboration did not consistently outperform advanced single models and that specialist methods remained stronger for medical visual question answering and structured-record outcome prediction [116]. A benchmark of generalist clinical agents reported non-significant accuracy gains of 0.5–8.9% at 10–100 times the token cost and approximately two to three times the latency [117]. In pathology, attention-based multiple-instance learning can localize evidence in a single forward pass [118]. These findings do not establish that agentic organization is inferior, but they show that interpretability, external generalization, and efficiency are not uniquely conferred by orchestration.

Agentic organization is most defensible when a case requires three properties. First, evidence must be assembled from heterogeneous sources that cannot be supplied to one validated model. Second, the next analytical step must depend on an intermediate result. Third, the output should expose evidence that a human can independently inspect. These properties motivate orchestration, but they do not guarantee faithful reasoning or clinical benefit.

Conversely, a validated specialist model is usually preferable for a bounded, single-modality task with a stable label space. Examples include metastasis detection, subtype classification, and quantitative immunohistochemistry. Such models can be simpler to validate and monitor, and may be faster or cheaper, although these properties must be measured rather than assumed. An orchestrating language model adds latency and a hallucination surface when the task requires neither branching nor external evidence. Mature deployments may therefore combine bounded specialist tools with orchestration only where case-dependent evidence assembly is necessary.

## Diagnostic applications: from pattern recognition to clinical reasoning

### Early screening and dynamic risk assessment

Diagnostic applications range from bounded screening tasks to exploratory systems that assemble multimodal evidence. The current evidence remains predominantly retrospective.

Deep learning systems have achieved pathologist-comparable performance for lymph-node metastasis detection [86], and human-AI collaboration can improve accuracy in selected settings [14, 119]. The colon-biopsy screening algorithm of Graham et al. [72] remains in Table 3 because its threshold is configured at deployment rather than selected by an inference-time reasoning process; it therefore meets none of D, T, or K. This classification concerns computational organization, not clinical value. The study provides interpretable feature engineering, systematic error analysis, and validation evidence that many agentic prototypes lack.

Across the pathology systems reviewed here, evidence is predominantly retrospective or benchmark based. Regulatory and systems-integration requirements for clinical agents are also still evolving [120]. Pathologists perform case-dependent triage, multiscale inspection, and comparison with prior material. Agentic systems could support parts of this process, but prospective studies must compare them with validated screening models and measure review burden, missed findings, and escalation behavior.

### Precise subtyping and grading via reasoning

Subtyping and grading represent a substantial proportion of pathology AI research, accounting for an estimated 30.9% of more than 1,400 publications in one review [121]. Fixed classifiers can perform strongly on bounded tasks but do not alter their inference graph in response to intermediate findings. Agentic systems may add value when cases require multiscale evidence assembly or tool selection. That value remains unproven without matched baselines and clinically relevant endpoints.

PathAgent [46] uses a fixed Navigator-Perceptor-Executor architecture. The Navigator uses PLIP [122] to select question-relevant regions, the Perceptor uses Patho-R1 [123] to describe morphology, and the Executor uses Qwen3 [124] to predict an answer, reflect, and request additional regions. On SlideBench-VQA [17], WSI-VQA [54], and PathMMU [89], it reported higher zero-shot VQA scores than WSI-LLaVA [55]. It also exceeded SlideChat [17] where that model reported results, namely SlideBench-VQA [17] and PathMMU [89]. Because baselines differed in their vision and language models, the reported margins cannot be attributed to agentic organization. Pathologist intervention increased system accuracy from 56.32% to 68.10%. The modules and tool set are fixed, while region selection, magnification, and reflection cycles vary by case. We therefore code D and T as Partial, K as No, and autonomy as Level 1–2. The study did not report a matched ablation that isolated reflection or its effect on final answers.

Agent-based biological simulation should not be conflated with agentic AI. Rema et al. [74] combined cell-level agent-based models, reaction-diffusion equations, and gradient boosting to model PDAC subclones. The study reported 92% sensitivity and 89% specificity under retrospective calibration. Its dynamic simulation does not select reasoning steps, tools, or external knowledge at inference, so Table 3 classifies it as non-agentic.

Strong specialist systems illustrate why architectural classification should be separated from performance. Innani et al. [73] benchmarked eight pathology foundation models for WHO 2021 glioma classification and reported external AUCs of 92.61–96.30%. Behzadi et al. [75] used a graph convolutional network for prostate cancer grading. Both follow predetermined inference graphs and are non-agentic under D/T/K, irrespective of predictive performance.

### Agent-driven multimodal integration in spatial pathology

Many pathology AI studies use H&E alone, while others already integrate molecular, radiological, or clinical data. The distinctive claim for agentic systems is not multimodality itself, but case-dependent selection and sequencing of tools. Spatial pathology, which preserves tissue context while measuring molecular features, is therefore a relevant test setting for adaptive orchestration.

TissueLab integrates pathology, radiology, and spatial-omics data through a modular tool factory [58]. In clinician-feedback experiments, the reported accuracy for colon tumour-cell identification increased from near zero to 94.9% after 10–30 minutes of interaction, while a prostate tumour-to-duct ratio task reached 99.8% accuracy after 2 minutes. These results concern interactive workflow adaptation on study tasks, not autonomous clinical validation.

Fixed multimodal fusion methods such as stKeep [76] follow a predefined feature-aggregation path. An agentic system could instead use intermediate findings to request another stain, perform spatial-neighbourhood analysis, or invoke a prognostic model. This potential should be distinguished from demonstrated benefit because most reviewed systems have not compared such branching with a matched fixed pipeline. Tables 2 and 3 summarize agentic and non-agentic methods, respectively.

## Prognostic applications: from statistical models to dynamic risk assessment

### From static prognostic models to adaptive orchestration

Prognostic modelling has progressed from histopathological and molecular stratification toward spatial and multimodal analysis of the tumour microenvironment [125, 126]. WSI systems now address classification [127], grading [128], benign-malignant discrimination [129], diagnosis [130], report generation [88], and visual question answering [54]. These capabilities do not by themselves make a system agentic. WSI-LLaVA [55] and SlideChat [17] use language interaction but retain fixed inference structures. Many end-to-end models also have limited case-specific adaptation to molecular or staging information [131, 132]. Orchestration could invoke different specialist tools as evidence changes [133], but its advantage over matched fixed multimodal models remains to be shown.

### Histopathology agents: from feature extraction to spatial reasoning

Specialist models already provide prognostic signals without agentic control. In 777 patients, single-slide AI grading achieved a C-index of 0.77 compared with 0.78 for pathologist review of whole prostatectomy specimens [134]. Related work associated morphological features with pathway activity, reports, and mRNA expression [135]. The PDAC framework of Rema et al. [74] models spatial subclonal dynamics but is an agent-based biological simulation, not a reasoning agent. Collectively, these studies motivate spatial and multimodal prognostic analysis but do not establish the superiority of agentic systems.

Kim et al. [77] used Lunit SCOPE IO to quantify TILs in 304 pancreatic-cancer specimens, defining inflamed, excluded, and desert phenotypes associated with overall survival. This is a fixed specialist workflow. CPathAgent [36] similarly uses a predefined navigation-reasoning pipeline and is coded as Partial for D, No for T, and No for K in Table 2. MUSK [79] is a multimodal foundation model evaluated retrospectively for melanoma recurrence, pan-cancer prognosis, and immunotherapy response; it is not an agent. An ARPA-H award supports the development of multimodal fusion tools intended to identify cancer-resistance traits and treatment-response biomarkers [136]; clinical validation has not been reported. These examples illustrate relevant component technologies rather than evidence of agentic clinical benefit.

### Hierarchical multi-agent architectures: novel strategies for cross-modal integration

Fixed multimodal fusion models have improved pan-cancer prognosis. CATfusion combined WSIs with five omics data types and reported an overall C-index of 0.668 across 31 TCGA cancers [80]. Brim reported a C-index of 0.682 across 12 cancer types [81]. Many such systems are trained by cancer type [137–139] and use predetermined fusion paths [140]. They cannot choose a different tool or knowledge source at inference, but this architectural limitation should not be conflated with poor predictive performance. Dependence on molecular tests that are not routine at every site may also limit deployment.

SurvAgent is a hierarchical multi-agent system for survival prediction [60]. It combines agents for multi-scale WSI analysis, gene-set processing, knowledge retrieval, and multimodal inference. Across five TCGA cohorts, it reported an overall C-index of 0.713 versus 0.706 for MOTCat [141]; comparisons with general-purpose models and MDAgent [142] or MedAgent [39] were larger, but those systems were not designed for the same modalities or endpoint [60]. The clinically relevant comparison is therefore the narrow margin against specialist fusion models. All five cohorts were derived from TCGA; limited demographic representation [143] and retrospective curation restrict transportability to contemporary routine practice. Ferber et al. [61] separately reported increased appropriate tool use and a higher proportion of expected answers supplied in a 20-case evaluation, although this remains a small proof-of-concept.

### Spatial omics agents: decoding tumor microenvironment heterogeneity

Spatially resolved analysis is relevant because tumour behaviour reflects both cancer-cell programmes and interactions with immune, stromal, and inflammatory components [144, 145].

Khan et al. [82] reported a proof-of-concept use of Claude 3.5 Sonnet on one Visium dataset, including spot-level gene-expression quantification with R^2^ = 0.86 and narrative immunological interpretation. The design was human directed and did not establish autonomous planning or causal pathway inference. Kim et al. [78] combined CT, pathology, and clinical variables in a fixed LLM-guided model for five-year lung-cancer survival, with external validation. Both studies show that language models can assist multimodal interpretation, but neither meets the operational D/T/K criteria for an agent.

More automated spatial-omics systems are beginning to appear. TissueLab [58] combines an LLM orchestrator, a tool factory, pathology images, and Visium HD data. It displays intermediate results and permits workflow editing. In tumour-cell proportion estimation, 10–30 minutes of clinician feedback increased reported accuracy from near zero to 94.9% and IoU from 10.1% to 88.9%. This proof-of-concept illustrates interactive adaptation but does not yet establish autonomous clinical performance.

STAgent [70] and PhenoGraph [71] extend this approach. STAgent links multimodal language models to spatial-analysis tools, whereas PhenoGraph selects and revises phenotype-analysis pipelines using biological knowledge graphs. These systems demonstrate automated task decomposition and tool selection in research workflows; prospective reproducibility and clinical utility remain untested.

These studies extend language-model-assisted interpretation toward systems that orchestrate multi-step spatial-omics workflows. Their principal contribution is workflow automation and integration. Clinical actionability remains unproven until results are reproduced across laboratories and assessed against prespecified biological or clinical endpoints.

### Cross-omics data integration and interpretability

Interpretability should be treated as an evaluation target, not an inherent property of agents. Ferber et al. [61] combined a pathology vision transformer, MedSAM [146], and OncoKB [147] within a tool-using oncology agent. In 20 realistic simulated cases, the system achieved 87.5% tool-selection accuracy and 91.0% correctness of clinical conclusions. Although this modular design makes tool boundaries inspectable, the study did not assess whether the generated rationales faithfully represented the underlying computation or improved experts’ ability to detect errors.

MEREDITH uses literature, guideline, trial, and summary agents for molecular-tumour-board support [62]. In 59 variant assessments it achieved 94.7% concordance with experts and all cited literature was verified in that evaluation. This does not establish that hallucinations are eliminated outside the tested cases. A separate hepatocellular-carcinoma framework [63] combined MRI, predicted pathology features, and clinical variables, then compared six independently evaluated LLM-based RAG decision modules for treatment recommendation.

Modularity, retrieval, verification loops, and human review can improve traceability, but they are architectural affordances rather than demonstrated interpretability. None of the reviewed systems quantifies whether rationales faithfully reflect the computation that produced the output or improve a pathologist’s ability to detect errors. Future evaluations should report the proportion of claims linked to specific image regions or retrieved passages, expert-confirmed support for those links, and the change in error-detection rate when rationales are shown. Tables 2 and 3 compare these properties across agentic and non-agentic systems.

**Evidence status of the prognostic systems reviewed.** Most systems in this section were developed using TCGA or single-institution retrospective cohorts. None has been evaluated prospectively against a prespecified clinical endpoint, and none has regulatory clearance for prognostic use. Reported concordance indices should therefore be interpreted as evidence of technical feasibility, not expected clinical performance. Table 2 records validation scope and publication status.

## Therapeutic guidance: intelligent decision support for precision medicine

**Fig. 3:**
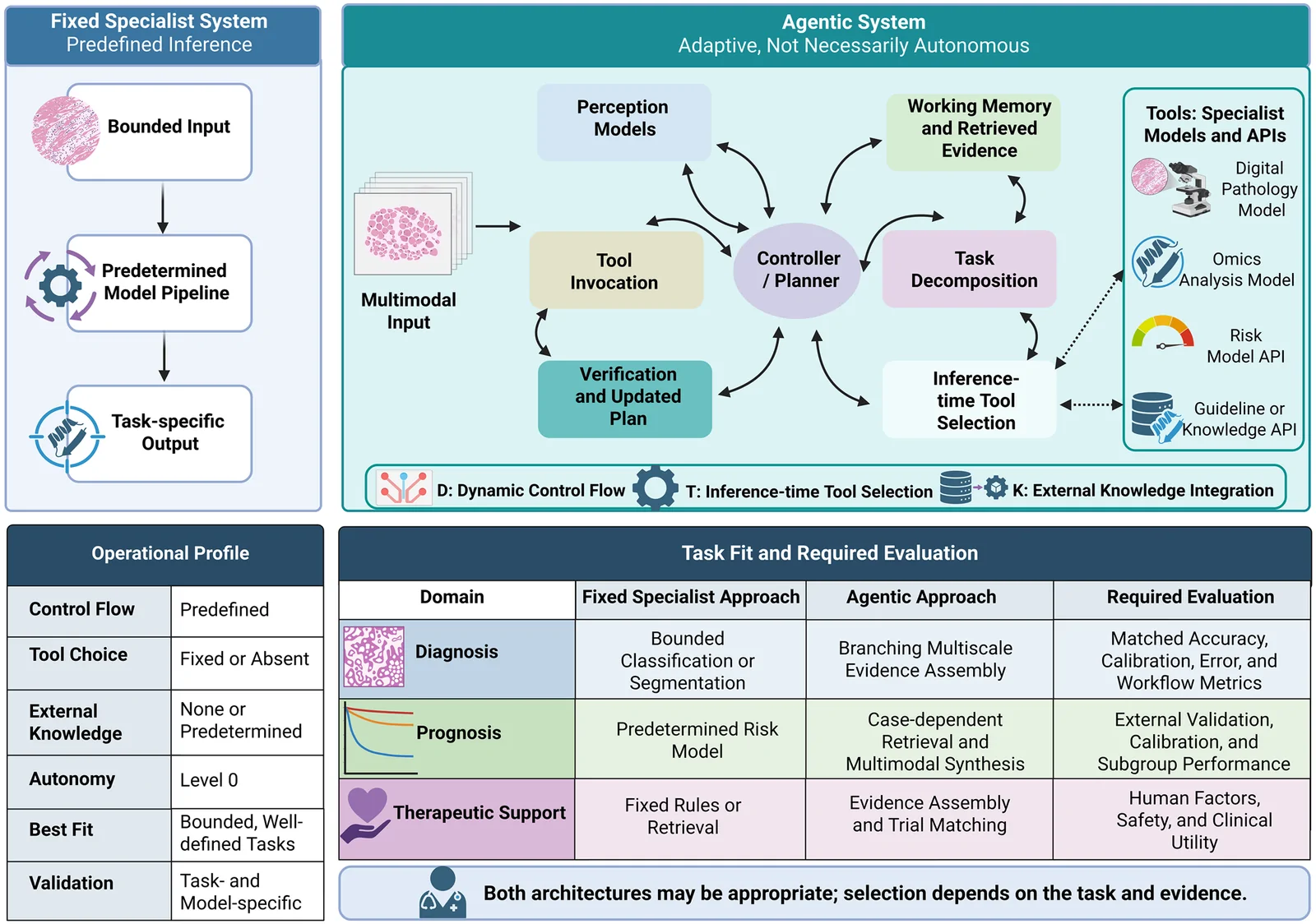
Operational comparison of agentic and non-agentic pathology systems. Compares fixed specialist systems with agentic systems using the operational criteria applied in this review. Fixed systems follow a predefined inference path and remain appropriate for bounded, well-defined tasks. Agentic systems may vary control flow, select tools at inference time, and integrate external knowledge when cases require evidence assembly or branching. The lower panel maps both approaches to diagnostic, prognostic, and therapeutic-support tasks and specifies matched evaluation requirements. Either architecture may be appropriate; architecture alone does not establish superior accuracy, interpretability, clinical readiness, or patient benefit. Created with BioRender.com

Therapeutic decision support requires integration of morphology, molecular biomarkers, clinical context, guidelines, and drug or trial information. Agentic architectures can coordinate these sources when the next analytical step depends on intermediate findings. Their value must, however, be established against simpler retrieval or specialist-model baselines and evaluated with clinically meaningful endpoints (Fig. 3).

### Closed-loop systems from pathology to therapeutic decision-making

Ferber et al. [61] evaluated a tool-using oncology agent that combined mutation prediction, image segmentation, and OncoKB retrieval. In 20 realistic simulated cases, the authors reported 87.5% tool-use accuracy, 91.0% correctness of clinical conclusions, and 75.5% guideline-citation accuracy. The proportion of expected answers supplied rose from 30.3% with GPT-4 alone to 87.2% with tools. This small simulated evaluation demonstrates technical feasibility, not clinical effectiveness.

### Pathomics-guided disease-specific treatment optimization

An HCC framework combined MRI-based prediction of seven pathological markers with six independently evaluated LLM-based decision modules and RAG from BCLC/CNLC guidelines [63]. The pathology-prediction stage reported an accuracy of 0.8902 for microvascular invasion. In a blinded retrospective evaluation of 105 cases, DeepSeek-R1 received the highest expert scores among the tested models for concordance, personalization, and feasibility. In a separate study, Morpheus [85] used counterfactual optimization of spatial-proteomics data to identify perturbations associated with T-cell infiltration and included in vitro validation. Neither study establishes patient benefit from agent-guided treatment selection.

### Baseline performance and limitations of general models in oncology treatment decisions

Ahthiane et al. [83] evaluated Claude 3 Opus, GPT-4 Turbo, and Llama 3 70B in 112 early-breast-cancer multidisciplinary-board cases, reporting appropriate recommendations in 86.6%, 85.7%, and 75.0%, respectively. Errors included outdated genomic-testing advice, weak surgical reasoning, and over-recommendation. These findings motivate, but do not prove the superiority of, systems with current guideline retrieval, specialist tools, and traceable evidence.

### Pathology feature applications in clinical trial matching

Loaiza-Bonilla et al. [66] reported a prospective operational evaluation of a neuro-symbolic multi-agent trial-matching system in 3,804 patients. The held-out cohort generated 23,912 candidate patient-trial pairs, of which 17,912 were confirmed by oncologists. The system achieved an F1 score of 0.82 (95% CI, 0.81–0.83), and median screening time fell from approximately 120 minutes of manual review to approximately 30 minutes, including automated processing and clinical review. This prospective study supports operational feasibility for trial matching, but it did not evaluate a pathology diagnostic endpoint or patient benefit. PRISM and OncoLLM [84] reported 63% criterion-level accuracy and a 35-fold cost reduction in retrospective trial matching. This local-deployment approach is not a prospective pathology workflow study.

### Agentic orchestration for multidisciplinary collaborative decision-making

The Microsoft Healthcare Agent Orchestrator [148] integrates agents for patient-history summarization, imaging, pathology, and clinical-trial matching. Its TBFact framework [65] reported 84% recall for high-importance information across 71 oncology cases. Publicly described evaluations remain early-stage, so this system should be regarded as an integration prototype rather than a clinically validated platform.

Across therapeutic-guidance studies, modular tools, retrieval, and multi-step reasoning can assemble evidence that is distributed across images, molecular reports, guidelines, and trial registries. The evidence base nevertheless consists mainly of small retrospective or benchmark evaluations. Reported performance should therefore be interpreted as technical feasibility, and each analytical tool, orchestration rule, and final recommendation requires separate validation. Tables 2 and 3 summarize the agentic and non-agentic systems discussed in this review.

## Challenges, ethics, and future directions

### Technical challenges

**Fig. 4:**
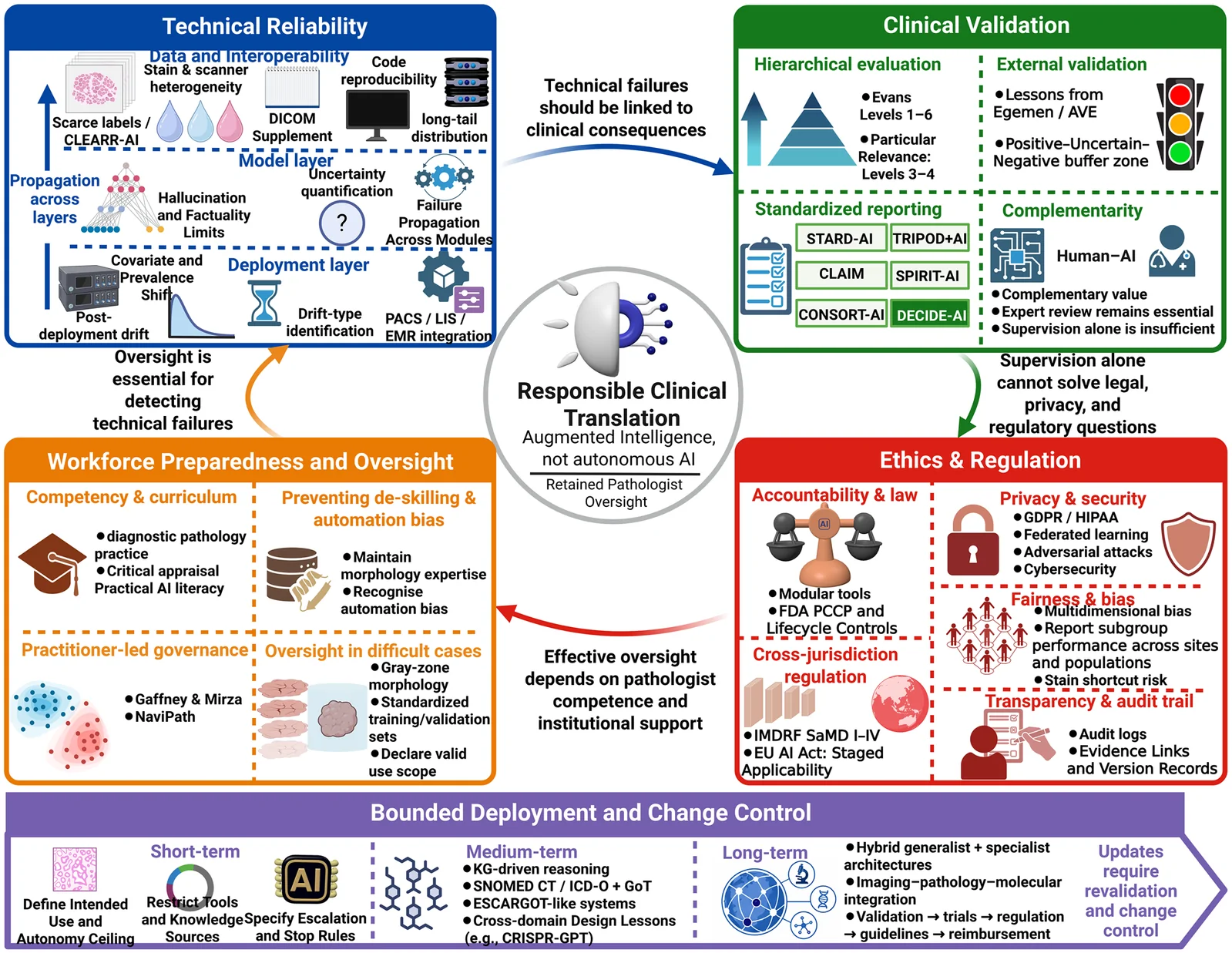
Translational requirements and research priorities for agentic systems in computational pathology. Summarizes five interdependent requirements for translation: technical reliability, clinical validation, regulation and ethics, workforce preparedness and oversight, and bounded deployment with change control. Technical failures must be linked to clinical consequences; governance must address accountability, privacy, security, fairness, traceability, and jurisdiction-specific requirements; and pathologists require the competence, authority, and institutional support to supervise use. The lower panel organizes short-, medium-, and long-term priorities and emphasizes that updates require revalidation and change control. The central principle is augmented intelligence with retained pathologist oversight. Created with BioRender.com

Clinical translation of agentic systems in computational pathology faces interdependent technical constraints (Fig. 4). Data quality and heterogeneity affect standardization and cross-site generalization. Hallucination, infrastructure incompatibility, and post-deployment drift persist even when upstream data and format issues are improved.

**Data-level obstacles.** Annotation quality, inter-observer variation, cross-institutional transfer, and image-quality heterogeneity remain central constraints [149]. CLEARR-AI provides a structured reporting framework for dataset objectives, curation, annotation, and quality review [150]. Staining, scanners, and local workflows can reduce generalization [103, 130]. Stain normalization can mitigate color variation but may introduce artifacts and cannot remove all domain shift [151]. Cross-site validation therefore also requires harmonized acquisition and metadata [152]. DICOM provides a vendor-neutral format and structured metadata for whole-slide images and annotations [153], although adoption remains incomplete. Reproducibility is also limited: a review of 160 studies found public code in 41 and independent external evaluation in only 16 of those 41 [154]. Rare tumours and atypical patterns create long-tail problems; in spatial transcriptomics, curriculum-learning “noisy” samples were enriched for biologically meaningful features [155]. Agents should therefore distinguish technical artifacts from rare but valid biology and report how such cases are handled.

**Model-level limitations.** Medical-image multi-agent systems often depend on empirical parameters and incur communication overhead [52]. Retrieval or knowledge graphs [100] and multi-agent verification [53] can reduce, but not eliminate, fabrication. In one clinical-documentation evaluation, 1.47% of statements were hallucinated and 44% of those hallucinations were major errors [156]. Even a low reported percentage cannot be interpreted as an acceptable residual risk in high-throughput clinical practice. Clinician surveys also report exposure to potentially harmful medical hallucinations [157]. General clinical agents have invented measurements and laboratory values [117], while pathology-specific risks include fabricated morphology, unperformed ancillary tests, and unsupported guideline claims. On MedHallu, the strongest reported F1 for difficult hallucinations was 0.625 [158]. Conformal prediction can bound error for fixed predictors under exchangeability and has reduced errors in AI-assisted prostate-biopsy assessment [159]. These guarantees do not cover an entire reasoning trajectory and weaken under distribution shift. Methods for factual language-model output [160] and set-valued generation [161] apply to claims or completed outputs, not every intermediate step. Calibration is therefore most defensible at specialist-tool outputs and final claims, with human review of the remaining trajectory.

**Engineering implementation.** In the FeTS challenge, deployment across 32 institutions required weeks, some models failed because of GPU incompatibility, and manual review found major annotation errors in 10.4% of test cases [162]. This experience illustrates that infrastructure heterogeneity, data quality, and coordination burden remain material even for mature segmentation systems.

**Computational cost and inference latency.** Pathology studies rarely report per-case latency, GPU-hours, or monetary cost. General clinical benchmarking found that agent orchestration used 10–100 times more tokens and approximately two to three times the response time for small, statistically non-significant accuracy gains [117]. These estimates are not directly transferable to gigapixel analysis, but they show that orchestration overhead must be measured.

Table 2 records only explicitly reported inference measurements. Training hardware is not treated as inference cost. Future studies should report model calls, hardware, end-to-end latency, regions processed, GPU-hours, and per-case monetary cost. Missing cost or latency reporting is treated as an evidence gap rather than neutral missing information.

Navigation exposes the same trade-off. Exhaustive tiling reduces omission risk but scales with slide area; heuristic navigation reduces computation by examining selected regions but may miss sparse findings. Existing agent benchmarks have not quantified this recall risk. Evaluation should therefore compare heuristic navigation with exhaustive-search reference standards in case sets enriched for isolated tumour cells, small foci of invasion, and rare mitoses.

**Post-deployment monitoring and model drift.** Input-level shift detection alone may not predict performance loss because a large covariate shift can be benign or harmful relative to a fixed decision boundary [163]. Roschewitz et al. [164] showed that confusing covariate with prevalence shift can worsen calibration after inappropriate recalibration. In a seven-hospital study of 143,049 patients, a label-agnostic pipeline detected harmful changes, and drift-triggered updating outperformed a locked model during COVID-19 (ΔAUROC 0.44, SD 0.02; *p* = 0.007) [165]. These findings, obtained outside agentic pathology, support ongoing monitoring and shift-specific remediation. They do not validate continual updating without change control.

### Clinical validation gap

Technical performance metrics alone are insufficient to establish clinical benefit or assess potential harm. Structured clinical validation is therefore required before practical deployment. Reis-Filho and Kather [166] identify five broad barriers to pathology AI translation: culture, data quality and bias, biomarker validation, regulation, and cost. In addition to these general barriers, agentic systems introduce questions about human factors, failure propagation across tools, and change control.

**Evaluation frameworks.** Evans et al. [167], drawing on radiology frameworks, propose six levels of evaluation: technical performance, diagnostic accuracy, effects on diagnostic thinking, effects on therapeutic decisions, patient outcomes, and socioeconomic outcomes. For agentic systems, the third and fourth levels are particularly relevant because they assess effects on clinical decisions rather than benchmark performance alone. The blinded multidimensional scoring and prompt-sensitivity analysis used in the HCC decision-support study [63], together with the TBFact framework [65], provide examples of early evaluation methods rather than validated clinical endpoints.

**Methodological insights from external validation.** Egemen et al. [168], in iterative optimization of the AVE AI system for cervical cancer screening, found that early algorithms with near-perfect internal-validation AUC failed on external datasets. The study’s positive-uncertain-negative strategy improved reproducibility for borderline cases by creating buffer zones. This strategy may inform evaluation of pathology agents: morphologically ambiguous lesions should be routed for further examination rather than forced into a binary class.

**Reporting frameworks can improve reproducibility.** TRIPOD+AI, STARD-AI, CLAIM, SPIRIT-AI, and CONSORT-AI address prediction models, diagnostic accuracy, imaging, and trials [169]. SPIRIT-AI and CONSORT-AI require reporting of input-data quality, clinical context, human-AI interaction, and error analysis [170]. DECIDE-AI focuses on early, small-scale evaluation of AI decision support as part of a human-machine system [171]. It is especially relevant to early clinical studies of pathology agents [171], but it does not replace later comparative trials.

Jin et al. [172] found that GPT-4 V answered 78.3% of questions missed by physicians in a closed-book experiment. Physicians’ ability to identify model reasoning errors correlated with their own expertise (Spearman ρ=0.917). This suggests complementarity, but also that human review is not a uniform safeguard. Oversight must therefore combine expert review with institutional accountability, data protection, and subgroup monitoring.

### Ethical and regulatory considerations

Section “Clinical validation gap” describes performance validation and the need for oversight. Safe and lawful deployment also depends on clear institutional responsibility, compliance across jurisdictions, and patient protection. The discussion below addresses four practical domains: accountability and legal frameworks, data privacy and security, fairness, and transparency.

**Accountability and regulatory frameworks.** Modular systems such as Ferber’s agent [61] and TxAgent [64] may allow individual tools to be validated separately, but responsibility for the combined workflow remains unresolved. In the United States, FDA guidance on predetermined change control plans and lifecycle management [173, 174] permits specified software modifications under prospective oversight. Internationally, IMDRF classifies medical-device software by the significance of its output and the severity of the clinical condition [175, 176], a framework adopted by the FDA [177] and broadened in final IMDRF guidance to medical device software [178]. In the European Union, AI-enabled devices subject to third-party conformity assessment may also fall within the high-risk AI framework [179, 180], with staged applicability [181]. A 2025 Commission proposal could alter that scope [182]. Because the regulatory position remains dynamic, agent updates to instructions, tools, or knowledge sources require documented change control and jurisdiction-specific assessment.

**Regulatory pathways beyond the United States and European Union.** The United Kingdom, Australia, Japan, and China regulate AI-enabled software through existing medical-device frameworks but differ in how intended purpose, adaptive change, premarket review, and postmarket oversight are handled. MHRA frames AI software across the product lifecycle. The TGA bases device status on intended purpose and requires approval for updates that alter purpose or performance. PMDA guidance addresses bias and postmarket learning in AI-based software as a medical device, whereas the NMPA has strengthened lifecycle classification, review, standards, and surveillance for AI-enabled devices [183–186]. Changes to an agent’s instructions, tool set, or retrieved knowledge base may therefore be regulated differently across markets. Multinational deployment requires a predefined change-control plan and jurisdiction-specific validation.

**Data privacy and system security.** GDPR and HIPAA require lawful processing, access control, and traceability for health data [187]. Federated learning can reduce direct data transfer, but a review of 15 computational-pathology studies [188] found that 13 were simulated and that domain alignment contributed more than aggregation refinements [188]. In a real multi-institutional study [189], network configuration took weeks and some training runs lasted up to 25 days. Local deployment, as used by PRISM [84], can reduce data egress and cost but does not remove model risk. Proof-of-concept adversarial attacks have changed high-confidence medical-image predictions with imperceptible perturbations [190]. Security risks are amplified by legacy imaging infrastructure and transferable attacks [191]. Clinical systems therefore require restricted interfaces, architecture-aware testing, patch management, and incident monitoring; no current defence is universally effective.

**Security risks specific to agentic architectures.** External retrieval and tool use create risks beyond those of a single-purpose classifier. PHI egress should be minimized through local execution, de-identification, and contractual restrictions. Retrieved text can also carry indirect prompt injection [192]; surveys of agent ecosystems identify weak validation at tool boundaries [193], and prompt injection is ranked among the leading risks for language-model applications [194]. Instructions and retrieved data should therefore be separated, tools should use least-privilege credentials, write actions should require human confirmation, and every invocation should be attributable. Role definitions and tool manifests may be disclosed to regulators without being published in full. Audit logs may themselves contain PHI and should be protected as clinical data while avoiding unnecessary retention of raw patient content.

Algorithmic bias can arise from demographic, geographic, molecular, technical, and workflow differences [195]. In pathology, site-specific H&E protocols may encode demographic information and enable shortcuts unrelated to disease morphology [195]. Across several WSI tasks, reported AUC gaps between White and Black patients ranged from 3.0 to 16.0 percentage points [196]. Self-supervised models reduced but did not eliminate these gaps, and standard mitigation assumptions may fail under cross-institutional shift [195]. Evaluation should therefore report subgroup performance with site, scanner, staining, and assay characteristics. Broader cohort diversity and transparent development practices may improve transportability [26, 27], but open-source release alone is not a fairness guarantee.

**Patient perspectives, consent, and liability.** In a nationally representative US survey, 62.7% considered notification of AI use very important; a single-centre medical-imaging survey reported 90.3% support for disclosure and 91.1% for an opt-out option [197, 198]. These estimates are context specific and do not establish a universal consent requirement. Pathology services should document clinically material AI involvement, communicate uncertainty through the treating team, and define any available opt-out pathway. US malpractice analysis indicates that physician conduct remains judged against the standard of care, which may complicate reliance on recommendations that depart from accepted practice [199]. Agentic systems should remain advisory, with pathologists retaining final sign-out authority.

**Transparency and auditability.** Reasoning traces do not by themselves demonstrate faithful interpretation. CASSIA generates quality scores and reasoning chains [200], and MEREDITH [62] achieved 94.7% expert concordance with verified citations in its evaluation. For clinical use, auditability also requires data lineage, model provenance, decision traceability, and compliance logging [201]. Source-verified architectures can link recommendations to retrieved evidence and record intermediate steps [201]. Regulatory requirements also address automated logs and electronic-record integrity [202]. Each inference should therefore record a stable model version and retain an audit record sufficient for review [203]. These mechanisms support drift investigation and accountability, but their effectiveness still depends on a pathologist able to interpret and challenge the output.

### Educational preparedness and the pathologist’s oversight role

The technical safeguards, validation frameworks, and governance structures discussed above depend on pathologists who can supervise, challenge, and override system outputs. AI-specific preparedness nevertheless remains uneven.

**Competency and curricular development.** Pathologists report interest in AI but also gaps in practical knowledge, while current residency curricula rarely define AI-specific entrustable activities [204]. Vos et al. [204] propose an activity covering model development, validity, generalizability, patient-context interpretation, human-AI limits, and ethical or legal issues. This addresses deskilling concerns reported by 7 of 16 interviewed pathologists [115]. Because error detection may depend on a clinician’s own diagnostic expertise [172], AI training should complement rather than replace sustained morphological education.

**Oversight structures and practitioner-led governance.** Gaffney and Mirza argue that practitioner involvement should shape development, explainability requirements, and limits on autonomous threshold changes [205]. Hassell et al. [206] note that models may perform well on common cases yet fail in morphological grey zones. They recommend standardized WSI validation resources, curated slide libraries, and explicit definition of the case spectrum for each tool. These measures support continuous education, escalation pathways, and human authority over deployment.

### Bounding the deployment envelope: closed-loop versus open-ended tasks

The preceding sections show that hallucination cannot be eliminated, that its clinical consequences in pathology are specific and potentially severe, and that available statistical safeguards address prediction sets rather than reasoning trajectories. The relevant question is therefore not whether agentic systems will become safe enough for primary diagnosis, but which pathology tasks permit case-level verification. We use the following stratification.

**Verifiable closed-loop tasks** have a predefined reference or verification procedure for the primary output. Examples include quantitative immunohistochemistry, mitotic counting, TIL density, lymph-node metastasis detection, structured report extraction, and eligibility matching from documented tumour features. Errors are not automatically detected, but their detection pathway and review cost can be specified. These tasks are the strongest candidates for near-term prospective evaluation under pathologist sign-out.

**Semi-verifiable tasks**, including grading, subtyping, and diagnostic categorization, rely on reference standards with inter-observer variation. A stratified output may be safer than forced classification. The positive-uncertain-negative design evaluated in cervical screening [168] illustrates how ambiguous cases can be routed for further examination rather than resolved without sufficient evidence.

**Open-ended generative tasks**, including free-text narrative reasoning, differential diagnosis without a bounded label space, and unconstrained treatment recommendation, often lack a bounded case-level verification procedure; independent expert re-derivation may be required. For this class, we do not consider the available evidence sufficient to support any role in which the system’s output is the primary determinant of a diagnostic conclusion. Benchmark performance on such tasks should be interpreted as evidence of capability rather than safety.

This stratification suggests a bounded design. An agent may perform verified closed-loop functions, assemble evidence without forcing a label in semi-verifiable tasks, and leave open-ended conclusions to human derivation. Modular functions can then be validated separately and governed through explicit change control. This architecture is more defensible than granting broad autonomous authority and attempting to constrain it after deployment.

### Future directions

The development of agentic pathology systems can be organized along a temporal horizon that maps onto the constraints identified above, progressing from near-term engineering refinements through knowledge-driven reasoning to longer-term architectural and translational goals.

**Short-term development.** Pathology-specific adaptation of multimodal foundation models, including PathAgent [46] and SurvAgent [60], should prioritize interface design, latency, calibrated uncertainty, and prospective external validation.

**Medium-term development.** Knowledge-graph reasoning should be adapted to pathology ontologies. ESCARGOT-like architectures [100] could combine SNOMED CT or ICD-O with dynamic retrieval, while lessons from CRISPR-GPT [207] suggest that task decomposition and tool augmentation may transfer across biomedical domains. These remain design hypotheses requiring pathology-specific evaluation.

**Long-term development.** Biomni demonstrates a broad biomedical action space [59], but breadth does not establish the depth required for nuanced pathology. A plausible architecture combines general planning with pathology-specific tools, criteria, and verification. The multiscale framework reviewed by Tarhini et al. [208] motivates an imaging-pathology-molecular design but does not validate it.

Clinical translation requires linked evidence from algorithm validation through clinical trials, regulatory review, guideline adoption, and reimbursement. Key barriers include trial design [166], regulatory-pathway selection [209], and uncertain payer value [210]. Priorities are multicentre prospective studies, lifecycle change control, real-world evidence, and interoperable standards. Benchmarks such as PathMMU [89] and WSI-VQA [54] support technical comparison but do not replace clinical utility studies tailored to agentic workflows.

Across all horizons, progress will depend less on isolated algorithmic advances than on the coordinated maturation of the data standards, validation paradigms, regulatory harmonization, and workforce competence that this section has shown to be mutually dependent.

## Conclusion

Agentic systems provide a distinct way to organize computational pathology workflows, but the evidence reviewed here establishes technical feasibility rather than clinical benefit. Most systems are retrospective prototypes, direct comparisons with matched non-agentic baselines are uncommon, inference costs are poorly reported, and prospective clinical endpoints are absent. Translation should therefore proceed task by task, beginning with verifiable outputs and requiring external validation, human-factors evaluation, and lifecycle governance before routine use.

The near-term objective is augmented rather than autonomous diagnosis. Pathologists should retain final sign-out authority, while systems expose the image regions, retrieved sources, tool outputs, and uncertainty supporting each claim. Research priorities include matched ablation studies, prospective multi-institutional evaluation, demographic-stratified reporting, measurement of review burden, and standardized interoperability. Progress should be judged by reproducible clinical utility and patient safety, not by architectural complexity alone.

## Data Availability

Not applicable.
